# Mokola Virus in Domestic Mammals, South Africa

**DOI:** 10.3201/eid1309.070466

**Published:** 2007-09

**Authors:** Claude T. Sabeta, Wanda Markotter, Debrah K. Mohale, Wonderful Shumba, Alexander I. Wandeler, Louis H. Nel

**Affiliations:** * Agricultural Research Council–Onderstepoort Veterinary Institute, Pretoria, South Africa; † University of Pretoria, Pretoria, South Africa; ‡ Canadian Food Inspection Agency, Nepean, Ontario, Canada

**Keywords:** Moloka virus, rabies-related, lyssaviruses, domestic mammals, South Africa, dispatch

## Abstract

We recently identified 2 Mokola viruses from domestic mammals (a dog and a cat) in South Africa. These cases occurred 8 years after the last reported case of infection with this virus. Our findings emphasize the endemicity of rabies-related lyssaviruses in South Africa and the need to better understand the epidemiology of Mokola viruses.

*Mokola virus* (MOKV) is classified as genotype (gt) 3 of the genus *Lyssavirus* in the family *Rhabdoviridae* (order Mononegavirales). Apart from MOKV, the genus *Lyssavirus* consists of 6 gts: classic rabies virus (gt1), Lagos bat virus (gt2), Duvenhage virus (gt4), European bat lyssavirus type 1 (gt5) and type 2 (gt6), and Australian bat lyssavirus (gt7). Some novel lyssaviruses identified in bat species in the former Soviet Union are considered putative gts within this genus (*1*).

Although gt1 viruses have a global distribution, gt5 and gt6 viruses are restricted to Europe and gt7 viruses are limited to Australia. Natural infections with gt2, gt3, and gt4 viruses have been found only in Africa. With the exception of MOKV, all lyssavirus gts and putative gts have been isolated exclusively or most frequently from chiropteran species. MOKV has never been isolated from these species, but only from terrestrial mammals. The first MOKV was isolated from shrews (*Crocidura* sp.) in Nigeria in 1968. Since then, >20 isolates of this lyssavirus have been found throughout Africa (Cameroon, Central African Republic, Ethiopia, South Africa, and Zimbabwe) (*2*–*12*) (Table 1).

**Table 1:** Moloka virus isolates identified in Africa

Location	Year of isolation	Species of origin	Reference
Ibadan, Nigeria	1968	Shrew (*Crocidura* sp.) (3 isolates)	(*2*)
Ibadan, Nigeria	1968	Human	(*3*,*4*)
Ibadan, Nigeria	1969	Shrew (*Crocidura* sp.)	(*3*)
Umhlanga Rocks, Kwazulu Natal Province, South Africa	1970 (identified in the 1980s)	Cat	(*12*)
Ibadan, Nigeria	1971	Human	(*3*)
Yaounde, Cameroon	1974	Shrew (*Crocidura* sp.)	(*5*)
Bangui, Central African Republic	1981	Rodent (*Lophuromys sikapusi*)	(*6*)
Bulawayo, Zimbabwe	1981	Dog (vaccinated) and cat (4 isolates)	(*7*)
Bulawayo, Zimbabwe	1982	Cat (2 isolates)	(*7*)
Addis Adaba, Ethiopia	1989−1990	Cat	(*8*)
Selous, Zimbabwe	1993	Cat	(*11*)
Mdantsane, Eastern Cape Province, South Africa	1995	Cat	(*9*)
East London, Eastern Cape Province, South Africa	1996	Cat	(*10*)
Yellow Sands, Eastern Cape Province, South Africa	1996	Cat (vaccinated)	(*10*)
Pinetown, Kwazulu Natal Province, South Africa	1997	Cat (vaccinated) (2 isolates)	(*10*,*12*)
Pietermaritzburg, Kwazulu Natal Province, South Africa	1998	Cat (vaccinated)	(*10*,*12*)
Nkomazi, Mpumalanga Province, South Africa	2005	Dog	This study
East London, Eastern Cape Province, South Africa	2006	Cat (vaccinated)	This study

We report the identification and characterization of 2 cases of infection with MOKV in South Africa. The first was in a domestic dog and is, to our knowledge, the first such case in South Africa. The second was in a domestic cat, the host species in which all previous isolates were found. The cat MOKV isolate belonged to 1 of 2 previously identified South African MOKV phylogenetic lineages, but the dog MOKV isolate appeared to have a different lineage not previously encountered in South Africa or elsewhere in Africa.

## The Study

In October 2004, a 3-month-old kitten (*Felis domesticus*) was adopted from the Society of the Prevention of Cruelty to Animals (East London, Eastern Cape Province, South Africa) and lived with its owner on a farm 23 km outside the city. It had been neutered and had been vaccinated at 10 months of age with an adjuvanted inactivated vaccine against rabies (Rabisin; Merial, Lyon, France), but no subsequent vaccinations were given. The cat spent most of the day indoors, but went out at night and returned in the morning. Unusual behavior was noticed in March 2006. It appeared dull and physically unbalanced and its pupils were dilated but it was not aggressive. The cat was humanely killed, and its brain was sent to the Onderstepoort Veterinary Institute for rabies testing.

On June 17, 2005, a 6-month-old puppy (*Canis familiaris*) was brought by its owner to a veterinarian in the rural town of Nkomazi (Mpumalanga Province, South Africa). The dog had a temperature of 39.8°C and no appetite. After symptoms were treated, the dog was discharged, but it was brought back 11 days later because it was paralyzed, dehydrated, and had a fixed stare. This animal had never been aggressive to other pets or humans. The dog was humanely killed, and its brain was sent to the Onderstepoort Veterinary Institute for rabies testing.

Direct immunofluorescent antibody test with an anti-rabies conjugate cross-reactive with African lyssaviruses showed numerous and strongly stained inclusion bodies in every field of impression smears of both brain samples. Isolation of virus was attempted by suckling mouse brain passage and cell culture (neuroblastoma cells; Diagnostic Hybrids, Athens, OH, USA); both methods were successful for the cat sample. However, neither method yielded an isolate from the dog sample, despite a lyssavirus-specific reaction in the original brain sample by direct immunofluorescent antibody test.

Subsequently, antigenic characterization was performed with a panel of 16 monoclonal antibodies to the nucleocapsid protein of rabies virus (Canadian Food Inspection Agency, Nepean, Ontario, Canada). Both samples showed reactivity patterns associated with MOKV (Table 2).

**Table 2:** Reactivity of virus isolates with 16 monoclonal antibodies to the nucleocapsid protein of rabies and rabies-related viruses*

Monoclonal antibody	Dog (gt1)	Mongoose (gt1)	Lagos bat (gt2)	Mokola (gt3)	Duvenhage (gt4)	MOKV404/05	MOKV173/06
26AB7	+	Var	–	–	–	–	–
26BE2	+	Var	–	–	–	–	–
38HF2 (positive control)	+	+	+	+	+	+	+
66–1C5 (negative control)	–	–	–	–	–	–	–
M1001	–	–	–	+	–	+	+
M1336	+	–	–	Var	–	–	–
M1349	Var	Var	–	Var	–	–	–
M1386	–	+	–	–	–	–	+
M1412	+	Var	–	–	–	–	–
M1494	Var	Var	–	–	+	–	–
M612	–	–	+	–	–	–	–
M837	–	–	–	–	+	–	–
M853	+	–	–	–	+	–	–
M856	+	–	–	–	+	–	–
M857	+	–	–	–	+	–	–
M879	+	–	–	Var	+	–	–

*gt, genotype; +, positive reactivity; Var, reactivity with some regional variants; –, negative reactivity.

Final confirmation of MOKV in both case samples was obtained by reverse transcription–PCR, nucleotide sequencing, and phylogenetic analysis as described (*12*). Phylogenetic analysis (Figure) showed that the virus isolated from the cat sample (designated MOKV173/06) belonged to the same lineage of MOKV isolates that were recovered from cats in the same region of South Africa (*12*). However, the virus detected in the dog sample (designated MOKV404/05) appeared to represent a different South African MOKV lineage that was phylogenetically positioned between known South African and Zimbabwean lineages. This MOKV had nucleotide similarities of 88.1%–90.4% and 85.3%–88.5% with viruses from Zimbabwe and South Africa, respectively.

**Figure:**
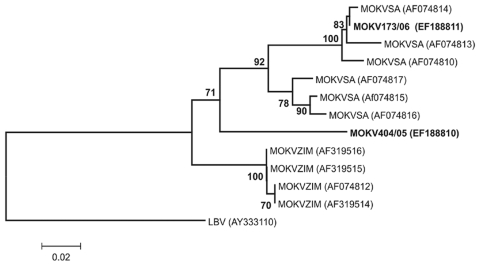
Phylogenetic tree based on 267 nt of partial nucleoprotein gene sequences of Moloka virus (MOKV) identified with the N1-N2 primer set as described (*12*). The tree shows phylogenetic positions of 2 recently identified cases of MOKV infection from South Africa (MOKV173/06 from a cat and MOKV404/05 from a dog) (in boldface) relative to previously characterized MOKV isolates from South Africa (SA) and Zimbabwe (ZIM) and Lagos bat virus (LBV) as the outgroup. GenBank accession nos. are shown in parenthesis. Bootstrap support values >70% are considered significant and indicated. Scale bar shows nucleotide substitutions per site.

## Conclusions

Infections with MOKV are rare; only 23 isolates are known. During the past 2 decades, all MOKV isolates have been found in South Africa. Because these viruses are not exclusive to South Africa (*2*–*12*), lack of isolates from other regions of Africa indicates a lack of active surveillance and limited diagnostic capabilities in many African laboratories. To our knowledge, the 2 cases of infection with MOKV we report are the first in 8 years from South Africa. These cases suggest that other cases may not have been recognized. Clinical signs in the dog and cat, including general neurologic manifestations with a lack of aggression, are often signs that warrant submitting samples for rabies testing.

We have identified regional variations in the antigenic composition of MOKV. Whether these variations are caused by neutral genetic drift or reflect different epidemiologic features is not known. Phylogenetically, divergence of these viruses into different lineages indicates active cycles and evolutionary changes that occur independently, but in close proximity (a few hundred kilometers apart).

Although the epidemiology of MOKV is incomplete, the case for a reservoir host(s) among small terrestrial animals of limited range is supported by our findings. Together with recent isolations of rabies-related lyssaviruses in a human (*13*) and wild animals (*14*,*15*), these reports emphasize the endemicity of these lyssaviruses in South Africa. Public health implications of African rabies-related lyssaviruses should be recognized by laboratory workers, researchers, veterinarians, wildlife personnel, gamekeepers, and pet owners. A better understanding of the epidemiology of these viruses is vital and can only be achieved by improved surveillance and awareness.
